# Insufficient blood supply of fovea capitis femoris, a risk factor of femoral head osteonecrosis

**DOI:** 10.1186/s13018-021-02564-6

**Published:** 2021-06-30

**Authors:** Keyang Zhao, Fangfang Zhang, Kun Quan, Bin Zhu, Guangyi Li, Jiong Mei

**Affiliations:** 1 Department of Orthopedic Surgery, Shanghai Jiao Tong University Affiliated Sixth People’s Hospital, Shanghai, 200233 China; 2 Tongji University School of Medicine, Shanghai, 200092 China; 3 Department of Orthopedics, The First Affiliated Hospital of Nanchang University, Nanchang, 330006 Jiangxi China; 4 Department of Orthopedics, The First Affiliated Hospital of Nanjing Medical University, Nanjing, 210029 China

**Keywords:** Nutrient foramen, Fovea capitis femoris, Traumatic osteonecrosis

## Abstract

**Background:**

A defective nutrient foramen in the fovea capitis femoris was hypothesized to reflect the blood circulation pattern of the femoral head, leading to insufficient blood supply and causing osteonecrosis of the femoral head.

**Methods:**

Normal and necrotic femoral head specimens were collected. The necrotic femoral head group was divided into a non-traumatic and traumatic subgroup. 3D scanning was applied to read the number, the diameter, and the total cross-sectional area of the nutrient foramina in the fovea capitis femoris. Chi-squared tests and independent t-tests were used to detect any differences in the categorical and continuous demographic variables. Logistic regression models were used to estimate the odds ratio (OR) for non-traumatic and traumatic osteonecrosis in different characteristic comparisons.

**Results:**

A total of 249 femoral head specimens were collected, including 100 normal femoral heads and 149 necrotic femoral heads. The necrotic femoral head group revealed a significantly higher percentage of no nutrient foramen (p < 0.001), a smaller total area of nutrient foramina (p < 0.001), a smaller mean area of nutrient foramina (p = 0.014), a lower maximum diameter of the nutrient foramen (p < 0.001), and a lower minimum diameter of the nutrient foramen (p < 0.001) than the normal femoral head group. The logistic regression model demonstrated an increasing number of nutrient foramina (crude OR, 0.51; p < 0.001), a larger total area of nutrient foramina (crude OR, 0.58; p < 0.001), a larger mean area of nutrient foramina (crude OR, 0.52; p = 0.023), a greater maximum diameter of the nutrient foramen (crude OR, 0.26; p < 0.001), and greater minimum diameter of the nutrient foramen (crude OR, 0.20; p < 0.001) significantly associated with reduced odds of osteonecrosis of the femoral head (ONFH). The necrotic femoral head group was further divided into 118 non-traumatic and 31 traumatic necrotic subgroups, and no significant difference was observed in any characteristics between them.

**Conclusions:**

Characteristics of the nutrient foramen in the fovea capitis femoris showed a significant defect of necrotic than normal femoral heads, and significantly reduced odds were associated with the higher abundance of the nutrient foramen in ONFH. Therefore, the condition of the nutrient foramen might be the indicator of ONFH.

**Supplementary Information:**

The online version contains supplementary material available at 10.1186/s13018-021-02564-6.

## Background

Osteonecrosis of the femoral head (ONFH) is a complex disease with ill-defined etiology and pathogenesis. About 70–80% of the patients with ONFH progress to secondary hip arthritis. Both operative and nonoperative treatments, including core decompression (CD) and other joint preserving treatments, have been performed and described with variable success rates [1–3]. Early diagnosis and combination treatment such as CD augmented with autologous bone grafting plus the implantation of bone marrow concentrate would be a benefit to reduce the pain and rate of total hip arthroplasty treatment [4].

The causes of ONFH include traumatic and non-traumatic. ONFH mainly occurs after direct trauma, such as femoral neck or head fracture, acetabular fracture, hip dislocation, severe sprain, or hip contusion. The pathogenesis of non-traumatic ONFH is unclear, and the main risk factors include systemic steroid administration [5–8], alcoholism [8, 9], stress disorder, sickle cell disease, and autoimmune diseases such as systemic lupus erythematosus (SLE) and antiphospholipid syndrome as well as local intravascular coagulation, bone marrow adipocyte hypertrophy, bone cell or endothelial cell apoptosis, and local intravascular hypercoagulability, which were due to chemotherapy, radiation therapy, cases’ disease, pancreatitis, and other factors.

ONFH was characterized by the death of osteocytes and the bone marrow and might be caused by the inadequate blood supply to the affected segment of the subchondral bone [10, 11]. Most of the femoral heads’ blood supply comes from the medial and lateral circumflex branches of the profunda femoris, and disrupted blood supply to the femoral head leads to ischemia and subsequent necrosis [12]. The vascular supply condition of the femoral neck is determined by the nutrient foramina and is mandatory for the safe execution of surgery around the hip junction [13–15]. Previous observational studies on the nutrient foramina in the human femoral head showed that the size and number of nutrient foramina in the fovea capitis femoris of the human femoral head vary greatly among patients and different regions [16–18].

Defects of fovea capitis femoris in the femoral head are the anatomical basis for the occurrence and development of a vascular necrosis of the femoral head. This concept has never been described and proven in the previous reports. For patients with femoral neck fractures, bleeding on the fracture surface reduces the blood supply to the femoral head and aggravates ischemia. For patients with avascular necrosis of the femoral head, lack of blood flowing into and out of the fovea of the femoral head aggravates the metabolic disorder of the femoral head. It is vital to understand the rules and changes of blood supply in evaluating and managing femoral neck fractures for personalized treatment. Complete information of the epidemiological and clinical characteristics is implemented for developing and establishing appropriate treatment strategies.

This study hypothesized that the defect of the nutrient foramen in fovea capitis femoris would reflect the blood circulation pattern of the femoral head, which would impact the repair of the blood supply under the action of risk factors, and then cause osteonecrosis of the femoral head. The nutrient foramen in the necrotic femoral head was compared with that in the normal femoral head to clarify the relationship between fovea capitis femoris and ONFH.

## Methods

This study collected femoral head specimens comprising intact normal femoral heads and necrotic femoral heads. The dry bone specimens of the femoral head were used as the normal femoral head group, which were obtained from our published study [18]. The specimens of femoral head necrosis were obtained from two tertiary teaching hospitals, including Shanghai Sixth People’s Hospital affiliated to Shanghai Jiao Tong University School of Medicine and Tongji Hospital Affiliated to Tongji University School of Medicine. The necrotic femoral head specimen was taken from the subjects with femoral head necrosis who were undergoing total hip replacement (THR) surgery and classified with the Steinberg classification system (a, b, and c subtypes). The bone surface was rinsed with normal saline, removed bloodstains, and fixed with formalin. These specimens were further divided into subgroups of non-traumatic osteonecrosis and traumatic osteonecrosis.

The femoral head surface was scanned using Micro-CT-100 (Scanco Medical, Bassersdorf, Switzerland) and output with a DICOM image for 3D surface reconstruction. The necrotic femoral head specimens with damaged fovea capitis femoris due to the collapse of the femoral head were excluded, and only femoral head with complete fovea capitis femoris and normal femoral head specimens were analyzed. The nutrient foramina in the fovea capitis femoris of the femoral head were analyzed on the 3D reconstruction specimens (Supplemental figure S1). Small holes on the smooth bone surface in the small depressions were first identified (Supplemental figure S2). After zooming in, nutrient foramina can be observed, like small holes deep in the bones (Supplemental figure S3). Through the section of the nutrient foramina, the continuous outer contour of the femoral head is interrupted at the nutrient foramina in the fovea capitis femoris of the femoral head. The bone surface nick at the interruption is a smoother transition rather than the sharp end of the fracture. The shape of the nutrient foramina is sometimes irregular, basically round and smooth. The number, diameter, and total cross-sectional area of the nutrition foramina were determined and recorded after the image is enlarged and confirmed as nutrient foramina, and the shorter diameter is chosen for measurement.

### Statistical analysis

Data were expressed as number (percentage) for categorical variables and mean ± SD for continuous variables. Baseline characteristics were compared between groups using chi-squared tests and independent t-tests to detect any differences in the categorical and continuous demographic variables. Logistic regression models were used to estimate the odds ratio (OR) and 95% confidence intervals (CIs) for non-traumatic and traumatic osteonecrosis in different characteristic comparisons. Data management and statistical analyses were conducted using SAS version 9.4 software (SAS Institute, Inc.). A two-sided P-value of < 0.05 was regarded as statistically significant.

## Results

A total of 249 femoral head specimens were collected, including 100 normal femoral heads and 149 necrotic femoral heads. The characteristics of nutrient foramina in the fovea capitis femoris of normal femoral heads and necrotic femoral heads are shown in Table 1. The necrotic femoral head group displayed a significantly higher percentage of no nutrient foramen (84.56% vs. 44%, p < 0.001), a smaller total area of nutrient foramina (0.30 ± 0.95 vs. 0.88 ± 1.20, p < 0.001), and a smaller mean area of nutrient foramina (0.16 ± 0.53 vs. 0.33 ± 0.48, p = 0.014) than the normal femoral head group. Besides, both the maximum diameter of the nutrient foramen (0.54 ± 0.55 vs. 0.18 ± 0.49, p < 0.001) and minimum diameter of the nutrient foramen (0.38 ± 0.41 vs. 0.14 ± 0.37, p < 0.001) are significantly larger in the normal femoral head group than in the necrotic femoral head group.

**Table 1:** Characteristics of nutrient foramina in the fovea capitis femoris of the normal femoral head vs. necrotic femoral head

Characteristic	Normal femoral head (N = 100)	Necrotic femoral head (N = 149)	P-value
Number of nutrient foramina			**< 0.001**
0	44 (44.00%)	126 (84.56%)	
1–3	36 (36.00%)	22 (14.77%)	
4–6	16 (16.00%)	0 (0.00%)	
> 6	4 (4.00%)	1 (0.67%)	
Area			
Total area of nutrient foramina, mm^2^	0.88 ± 1.20	0.30 ± 0.95	**< 0.001**
Mean area of nutrient foramina, mm^2^	0.33 ± 0.48	0.16 ± 0.53	**0.014**
Diameter			
Maximum diameter of the nutrient foramen, mm	0.54 ± 0.55	0.18 ± 0.49	**< 0.001**
Minimum diameter of the nutrient foramen, mm	0.38 ± 0.41	0.14 ± 0.37	**< 0.001**

Data are presented as mean ± SD or n (%). Significant values are shown in bold

The logistic regression model demonstrated that increasing number of nutrient foramina (crude OR, 0.51; 95% CI, 0.40–0.66; p < 0.001), larger total area of nutrient foramina (crude OR, 0.58; 95% CI, 0.44–0.77; p < 0.001), larger mean area of nutrient foramina (crude OR, 0.52; 95% CI, 0.30–0.92; p = 0.023), greater maximum diameter of the nutrient foramen (crude OR, 0.26; 95% CI, 0.15–0.45; p < 0.001), and greater minimum diameter of the nutrient foramen (crude OR, 0.20; 95% CI, 0.10–0.42; p < 0.001) were significantly associated with reduced odds of ONFH (Table 2). The multivariate regression analysis was not performed due to the multicollinearity among all the independent variables (data not shown).

**Table 2:** Univariate odds ratios of different characteristics for the necrotic femoral head

Characteristic	Univariate OR (95% CI)	P value
Number of nutrient foramina	0.51 (0.40–0.66)	**< 0.001**
Area		
Total area of nutrient foramina, mm^2^	0.58 (0.44–0.77)	**< 0.001**
Mean area of nutrient foramina, mm^2^	0.52 (0.30–0.92)	**0.023**
Diameter		
Maximum diameter of the nutrient foramen, mm	0.26 (0.15–0.45)	**< 0.001**
Minimum diameter of the nutrient foramen, mm	0.20 (0.10–0.42)	**< 0.001**

Significant values are shown in bold

*Abbreviations*: *OR*, odds ratio

The 149 necrotic femoral heads were further divided into two subgroups, including 118 non-traumatic and 31 traumatic necrotic femoral heads. Characteristics of nutrient foramina in the subgroup of traumatic osteonecrosis were also studied to delineate further the association between nutrient foramina and traumatic osteonecrosis (Table 3). However, there appeared to be no significant difference in any characteristics between the two subgroups.

**Table 3:** Characteristics of nutrient foramina in the fovea capitis femoris of non-traumatic osteonecrosis vs. traumatic osteonecrosis of the femoral head

Characteristic	Non-traumatic osteonecrosis (N = 118)	Traumatic osteonecrosis (N = 31)	P-value
Number of nutrient foramina			0.142
0	102 (86.44%)	24 (77.42%)	
1–3	16 (13.56%)	6 (19.35%)	
4–6	0 (0.00%)	0 (0.00%)	
> 6	0 (0.00%)	1 (3.23%)	
Area			
Total area of nutrient foramina, mm^2^	0.23 ± 0.85	0.59 ± 1.22	0.131
Mean area of nutrient foramina, mm^2^	0.15 ± 0.54	0.22 ± 0.47	0.481
Diameter			
Maximum diameter of the nutrient foramen, mm	0.15 ± 0.46	0.29 ± 0.56	0.171
Minimum diameter of the nutrient foramen, mm	0.12 ± 0.36	0.21 ± 0.42	0.248

Data are presented as mean ± SD or n (%)

## Discussion

A significant difference in nutrient foramina characteristics prevailed between normal and necrotic femoral heads, including the percentage of no nutrient foramen (44% vs. 86%), total area, mean area, maximum diameter, and minimum diameter of the nutrient foramen. The defect of nutrient foramina in necrotic femoral heads was significantly higher than the normal. Among 31 specimens of traumatic femoral head necrosis, 77.4% had no nutrient foramina. Besides, the abundance of nutrient foramen was significantly associated with low odds of ONFH. The defect of nutrient foramina at the femoral head could result from vascular foramina defect in this area, suggesting a clear association between the nutrient foramina and ONFH.

The avascular necrosis of the femoral head has been reported as a type of osteonecrosis due to disruption of blood supply to the proximal femur and can occur due to various causes, either non-traumatic or traumatic in origin [12, 14]. The blood supply to the head of the femoral head comes from both the artery of the ligamentum trees and the medial and lateral circumflex branches of the profunda femoris [12]. Limited collateral circulation was also reported to disrupt the blood supply to the femoral head, leading to ischemia and subsequent necrosis. If restoration of blood supply does not occur promptly, this will lead to the progressive death of osteocytes followed by the collapse of the articular surface and eventually by degenerative arthritis [19]. Impaired blood flow might lead to defective angiogenesis, osteogenesis, and downregulation of Notch signaling in endothelial cells, observed in murine long bones [20].

Kusumbe et al. reported that the role of type H vessels in bone formation is revealed [21]. Type H vessels are a new subtype of capillary vessels with different morphological, molecular, and functional properties. Type H vessels are distributed on the surface of the endosteum and metaphyses. The proportion of Type H endothelial cells in bone endothelial cells is deficient (1.77%); however, the mRNAs of growth factors such as PDGFa, PDGFb, Tgfb1, Tgfb3, and Fgf1 a are highly expressed, which can thereby affect osteoblasts survival and proliferate as well as promote the selective distribution of osteoblast precursor cells Type H vessels are involved in angiogenesis, which has high proliferation ability and can produce L-type endothelial cells. Type H vessels affect the growth of the bone vasculature and generate distinct metabolic and molecular microenvironments to stabilize endothelial cells around blood vessels, thus coupling osteogenesis and angiogenesis [21]. Therefore, the presence of nutrient foramina in the femoral head might cause differences in the blood flow pattern of the femur. When femoral head fractures occur, changes in nutrient foramina affect the activity of endothelial cells and change the blood supply of the femoral head, compensating its ability. When the blood of the femoral head flows through the superior, inferior, anterior retinacula, and ligamentum teres artery, these four blood vessels can be connected in the femoral head. Once there are no nutrient foramina for the ligamentum teres artery to enter and flow out, the entire femoral head circulation will become a closed condition. When the femoral neck is fractured, blood flow can only enter and leave the femoral head through the superior and inferior retinacula artery. At this time, the hemodynamics of the femoral head is changed and affecting blood flow, blood flow resistance, and pressure in the capillary.

Displaced femoral neck fracture patients were reported to have a higher risk of vascular necrosis than non-displaced femoral neck fracture ones, which might be due to the blood supply to the femoral head is disrupted after displacement [22]. However, not all patients with severely displaced femoral neck fractures will suffer from ONFH in clinical practice [23]. Besides, one patient with femoral neck fractures with failed internal fixation was reported to obtain well prognostic results and did not reveal any evidence of avascular necrosis of the femoral head [24]; this suggests that the ONFH might not be caused by only the femoral neck fractures. In this study, necrotic femoral head specimens were further analyzed to see any different nutrient foramina patterns, and the result showed no significant difference in any characteristics between the subgroup of non-traumatic and traumatic necrotic femoral head specimens. It could reveal that the traumatic cause might not be the risk factor of ONFH.

An osteotomy was a treatment for ONFH by changing the spatial position of the necrotic portion of the femoral head. However, Quaranta et al. have reported that approximately one-third of the osteotomies performed in cases of ONFH were converted to THR over 7 years [25]. The information of nutrient foramina in the fovea capitis femoris mentioned in this study might be used to define the parameters of the patient and improve the outcome of the osteotomy technique.

With the assistance of high-resolution CT scanning, information about the presence of nutrient foramina in the fovea capitis femoris might be expected as one of the monitoring indicators of ONFH risk. For the elderly lacking nutrient foramina in the fovea capitis femoris, artificial hip replacement would be suggested. For the young and middle-aged patients suffering from femoral neck fractures, drilling from the greater trochanter, the neck of the femur, and the fovea capitis femoris combined with internal fixation is recommended. For patients with femoral neck fractures and lacking nutrient foramina in the fovea capitis femoris, or patients with non-traumatic ONFH, this might be a treatment option. This anatomical phenomenon enriches the etiology of ONFH and provides an alternative strategy for the individualized treatment options for femoral neck fractures, especially the internal fixation treatment of femoral neck fractures in young people.

## Conclusion

A significant defect of the nutrient foramen in the fovea capitis femoris of necrotic femoral heads was observed in this study, and a higher abundance of nutrient foramen was significantly associated with reduced odds of ONFH. Therefore, the information from high-resolution CT scanning relating to the nutrient foramina in the fovea capitis femoris might be a monitoring indicator of the ONFH.

## Supplementary Information

**Additional file 1: Supplemental Figure S1**. The 3D reconstruction picture of the femoral head specimen shows the nutrient foramina in the fovea capitis femoris of the femoral head.

**Additional file 2: Supplemental Figure S2**. The zoom-in picture presents the fovea capitis femoris of the femoral head.

**Additional file 3: Supplemental Figure S3**. The enlarged nutrient foramina, indicating that the nutrient foramina penetrate the bone, and the contour of the cancellous bone in the femoral head can be observed from the nutrient foramina.

## Data Availability

The datasets used during the current study are available from the corresponding authors on reasonable request.

## Abbreviations

ONFH: Osteonecrosis of the femoral head
SLE: Systemic lupus erythematosus
OR: Odds ratio
CIs: Confidence intervals
