# Supplementary figures and images for: Insufficient blood supply of fovea capitis femoris, a risk factor of femoral head osteonecrosis
Source: J Orthop Surg Res. 2021 Jun 30;16:414. doi: 10.1186/s13018-021-02564-6 (PMC8243432; doi:10.1186/s13018-021-02564-6)

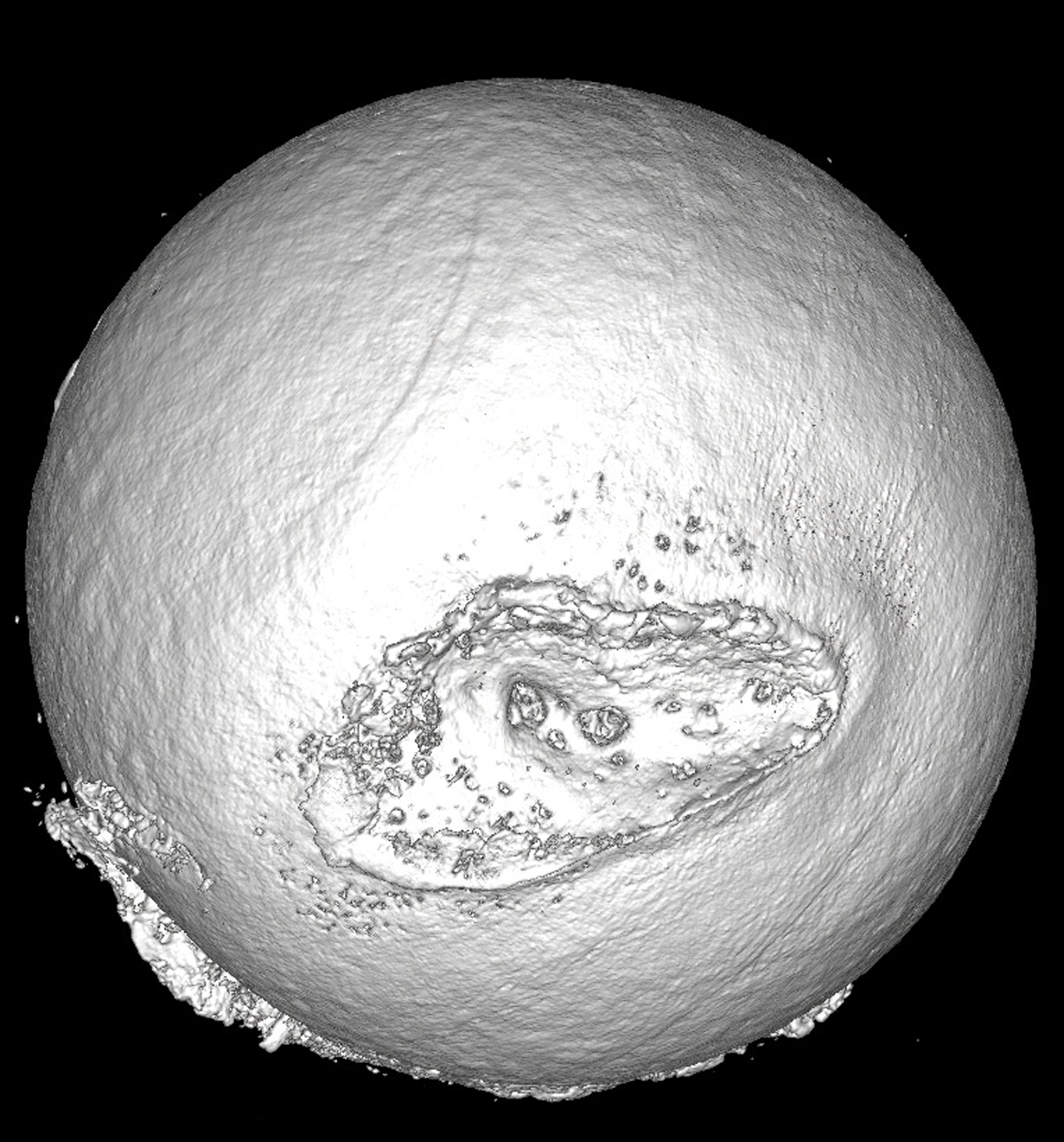

Supplement: Supplementary file 1 — Additional file 1: Supplemental Figure S1. The 3D reconstruction picture of the femoral head specimen shows the nutrient foramina in the fovea capitis femoris of the femoral head. [file 13018_2021_2564_MOESM1_ESM.tif]

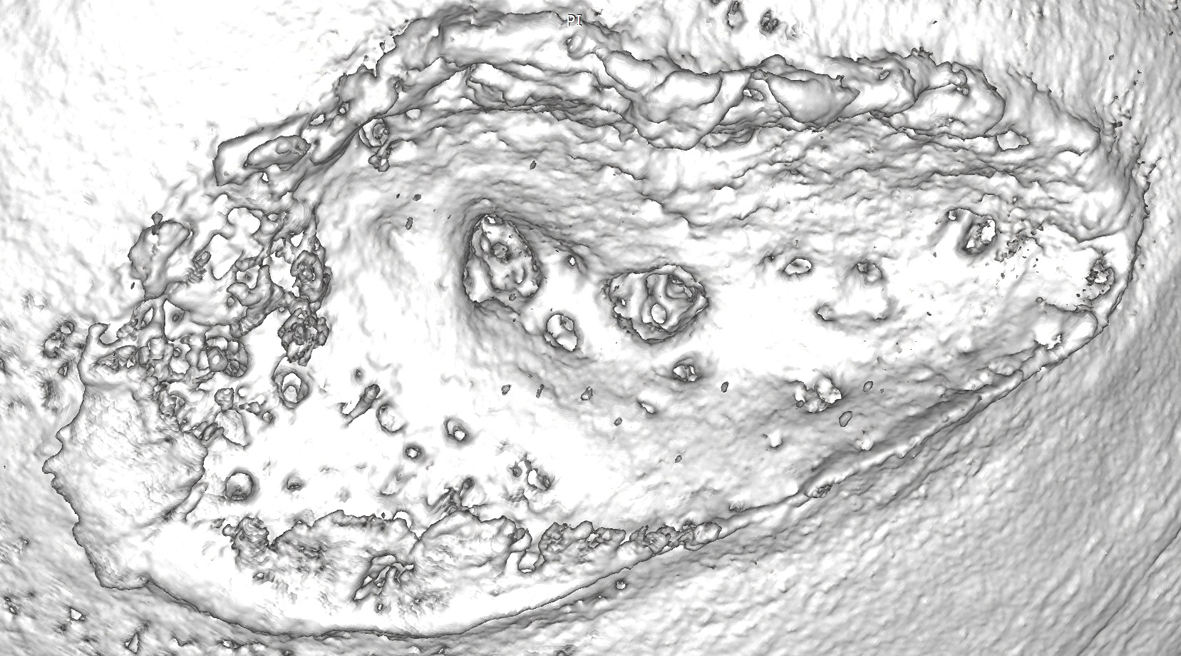

Supplement: Supplementary file 2 — Additional file 2: Supplemental Figure S2. The zoom-in picture presents the fovea capitis femoris of the femoral head. [file 13018_2021_2564_MOESM2_ESM.tif]

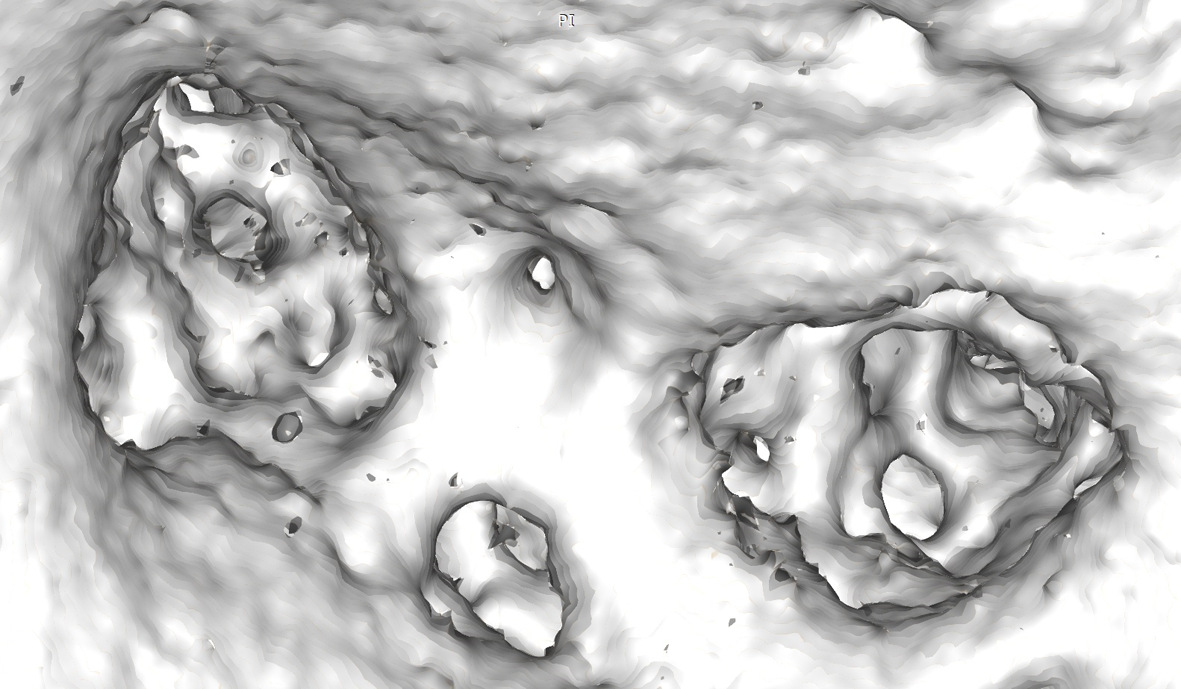

Supplement: Supplementary file 3 — Additional file 3: Supplemental Figure S3. The enlarged nutrient foramina, indicating that the nutrient foramina penetrate the bone, and the contour of the cancellous bone in the femoral head can be observed from the nutrient foramina. [file 13018_2021_2564_MOESM3_ESM.tif]
